# Shortening Duration of Swine Exhibitions to Reduce Risk for Zoonotic Transmission of Influenza A Virus

**DOI:** 10.3201/eid2810.220649

**Published:** 2022-10

**Authors:** Dillon S. McBride, Jacqueline M. Nolting, Sarah W. Nelson, Michele M. Spurck, Nola T. Bliss, Eben Kenah, Susan C. Trock, Andrew S. Bowman

**Affiliations:** The Ohio State University, Columbus, Ohio, USA (D.S. McBride, J.M. Nolting, S.W. Nelson, M.M. Spurck, N.T. Bliss, E. Kenah, A.S. Bowman);; Centers for Disease Control and Prevention, Atlanta, Georgia, USA (S.C. Trock)

**Keywords:** influenza A virus, swine, viruses, viral zoonoses, exhibitions, respiratory infections, zoonoses, vaccine-preventable diseases, United States, influenza

## Abstract

Reducing zoonotic influenza A virus (IAV) risk in the United States necessitates mitigation of IAV in exhibition swine. We evaluated the effectiveness of shortening swine exhibitions to <72 hours to reduce IAV risk. We longitudinally sampled every pig daily for the full duration of 16 county fairs during 2014–2015 (39,768 nasal wipes from 6,768 pigs). In addition, we estimated IAV prevalence at 195 fairs during 2018–2019 to test the hypothesis that <72-hour swine exhibitions would have lower IAV prevalence. In both studies, we found that shortening duration drastically reduces IAV prevalence in exhibition swine at county fairs. Reduction of viral load in the barn within a county fair is critical to reduce the risk for interspecies IAV transmission and pandemic potential. Therefore, we encourage fair organizers to shorten swine shows to protect the health of both animals and humans.

Swine are critical hosts for influenza A viruses (IAV) because they can be co-infected with IAV from multiple host species (*1*). Swine also have close proximity with humans through agricultural interfaces, making them a focus as a reassortment vessel for IAV with pandemic potential (*1*,*2*). The origin of the 2009 influenza A(H1N1) pandemic is attributed to North American swine, highlighting the effect of this host species on novel IAV emergence (*3*–*5*). 

Swine-origin IAV that infects humans, also known as variant IAV, is reportable to the Centers for Disease Control and Prevention in the United States. Since 2011, >475 confirmed variant IAV cases have been reported in the United States (*6*). Most of these variant cases have reported swine contact at agricultural fairs before infection (*7*–*13*). At local county fairs, hundreds of pigs may commingle in the same barn for >1 week. When only a few pigs arrive at the fair infected with IAV, ample time has passed by the end of the fair for viral amplification, and we detected very high prevalence in the swine (*14*,*15*). The time afforded for infection to spread throughout exhibition swine within a single fair, probably drives the high viral load in the barn, which can increase the risk for zoonosis.

In the United States, although commercial swine operations often have a higher level of biosecurity than their exhibition swine counterparts, diverse lineages of IAV originating from commercial swine are annually found in exhibition swine (*16*,*17*). These viruses move with the exhibition swine as they travel within complex networks of shows across the country (*13*,*18*). This commingling with hundreds to thousands of other pigs leads to viral reassortment, dissemination, and ultimately interspecies transmission of IAV (*13*,*18*). This system provides a unique human–animal interface as a conduit for zoonotic emergence of IAV.

Variant cases of IAV are most often reported in association with fairs that have very high IAV prevalence within their exhibition swine population (*8*,*9*). Accordingly, zoonotic transmission mitigation strategies should target reduction of IAV prevalence in swine. Previous risk assessment for IAV mitigation at agricultural fairs had shown that exhibitions with >200 pigs had a greater risk for having IAV-positive pigs; therefore, fewer pigs commingling in the barn together at any one time was hypothesized to decrease the prevalence of viral shedding, but few other risk factors were identified (*19*).

In an effort to prevent zoonotic transmission from occurring between swine and humans at agricultural exhibitions, the Swine Exhibitions Zoonotic Influenza Working Group, consisting of animal and public health officials, drafted Measures to Minimize Influenza Transmission at Swine Exhibitions, 2013 (*20*), which has been updated in subsequent years. The measures are divided into practices for use by exhibition organizers and exhibitors before, during, and after the swine exhibition period. Those measures include, but are not limited to, becoming familiar with clinical signs of illness in both pigs and humans, reporting observed clinical signs to the proper authorities, practicing common hygiene practices (e.g., handwashing), using a 7-day downtime between swine exhibitions, and shortening the duration of exhibitions to 72 hours. Recommendations, including the 72-hour recommendation, had been outlined previously by the Indiana State Board of Animal Health (*21*). Although those practices are based on common public and animal health theories, most of the measures were not based on existing scientific evidence for preventing swine-to-human IAV transmission.

We sought to evaluate the recommendation to limit swine exhibitions to <72 hours. During 2014 and 2015, we conducted daily IAV testing at 8 agricultural fairs in the United States, in which we sampled all exhibition swine every day to measure changes in prevalence longitudinally during the exhibitions. We then evaluated IAV in swine at agricultural fairs that had implemented the shortened, 72-hour recommendation for swine exhibitions compared with fairs that did not during 2018 and 2019.

## Materials and Methods

### Longitudinal Study (2014 and 2015)

We enrolled 8 agricultural fairs (4 in Ohio and 4 in Indiana) to participate in the study on the basis of 3 factors: >350 swine typically being exhibited; previous IAV recovery in pigs, humans, or both associated with the exhibition; and the fair organizer’s willingness to participate. Investigators coordinated with each fair organizer to determine the most accommodating schedule to collect samples at 24-hour intervals. The sample collection start time varied across the exhibitions in relation to hours after arrival of swine.

We collected nasal wipes from swine as previously described and in accordance with The Ohio State University Institutional Animal Care and Use Committee protocol (no. 2009A0134) (*22*,*23*). We recorded individual pig identification numbers. Origin time for each fair corresponds with the first time pigs could be identified, were required to be in place in the swine barn, or both. Four of the sampled fair events (fairs A and B in 2014 and 2015) included a weigh-in event shortly after the swine arrived on the fairgrounds. Origin time for those fairs is the weigh-in time and for all other fairs is the arrival deadline for swine. We preserved samples on dry ice for transportation to the laboratory, where we kept them in long-term storage at −80°C.

We screened samples collected from the first and last days of the exhibitions with real-time reverse transcription PCR (rRT-PCR). We used Mag-Bind Viral DNA/RNA (Omega Bio-Tek, Norcross, https://www.omegabiotek.com) according to manufacturer’s protocol for RNA extraction. We also used DiaControlRNA (Diagenode Diagnostics, https://www.diagenodediagnostics.com) as an internal positive control to ensure validity of our extraction and PCR. We used National Veterinary Services Laboratory PCR primer protocol (no. SOP-BPA-9034.04) with SuperScript One-Step RT-PCR (Invitrogen, https://www.thermofisher.com) according to the manufacturer’s protocol. If samples collected on the first or last days of the fair were positive for IAV, we screened all remaining samples by using the same technique. We completed virus isolation attempts on select rRT-PCR–positive samples collected on the first and last day of individual exhibitions, as previously described (*14*). Any fairs from which we were not able to isolate any IAV in MDCK cell culture we considered to be negative. 

One fair (2015 D) had 5 rRT-PCR–positive samples, none of which resulted in successful culture of IAV. We attributed these rRT-PCR positives to probably be carryover from previous infection or contamination from home-farm environment and not reflective of a productive IAV infection in the pigs at that fair. 

We calculated the estimated hazard of IAV infection by using the time from the origin of each fair until the detection of IAV infection or censoring in each pig. We did not consider swine to be at risk until the first sampling after weigh-in for fairs that included a weigh-in as described previously. For each fair, we smoothed the increments of the Nelson–Aalen cumulative hazard estimate by using an Epanechnikov kernel function, which is the default in Stata version 14 (StataCorp LLC, https://www.stata.com).

### 72-hour Recommendation (2018 and 2019)

After our longitudinal study in 2014 and 2015, some fairs began to adopt the recommendation to reduce swine shows to <72 hours. As a part of our ongoing surveillance program in 2018 and 2019, we collected >20 nasal samples per fair as described previously (*15*,*22*). We collected samples from exhibition swine at 195 individual fair events on the last day of the fair and selected pigs for sampling to evenly represent all spatial areas of the barn. This sampling scheme was designed to maximize detection of any IAV present in exhibition swine, which primarily have subclinical infections (*15*). We tested samples for IAV with the VetMAX‐Gold SIV Detection rRT-PCR Kit (Life Technologies, https://www.thermofisher.com) and conducted virus isolation (MDCK cells) to estimate prevalence at each fair as previously described (*18*). We used virus isolation to estimate prevalence at these fairs because we expected it to reflect animals actively shedding IAV, which is the risk factor of concern regarding interspecies transmission. We calculated swine exhibition duration on the basis of the number of days between arrival and our sample collection. We classified levels of implementation into 3 categories: fairs that did not reduce duration to <72 hours, fairs that released any portion of their pigs at <72 hours, and fairs that released or sold all swine before 72 hours. To evaluate differences in IAV prevalence between the different 72-hour implementation categories, we used a Kruskal–Wallis test followed by Dunn’s test for pairwise comparisons, applying the Benjamini–Hochberg method to control the false-discovery rate. We used Stata version 14.2 for statistical analyses.

## Results

### Longitudinal Surveillance within Fairs

During 2014 and 2015, we collected 39,768 nasal wipes from 6,768 individual pigs exhibited at 8 agricultural fairs (16 total fair events) (Table 1). We collected samples from all pigs present on each day of the fair; however, not all pigs remain present for the full duration of the fair. Many pigs were removed before the formal end of the fair, resulting in fewer pigs still at the fair on the last day compared with the beginning (Appendix Table 1). On the basis of fair structure, we expected this right censoring would be unrelated to health status of individual pigs. 

**Table 1:** Longitudinal sampling efforts of swine at 16 agricultural fairs included in study of IAV transmission, Ohio and Indiana, USA, 2014 and 2015*

Fair	2014				2015		
	No. swine exhibited at fair	No. samples collected	HA–NA subtypes		No. swine exhibited at fair	No. samples collected	HA–NA subtypes
Fair A	377	1,927	H1N1, H3N2, mixed		400	2,092	H1N2, H3N2, mixed
Fair B	424	2,741	H1N1, H3N2, mixed		414	2,719	H3N2
Fair C	274	1,200	Negative		281	1,233	Negative
Fair D	367	2,858	Negative		349	2,732	Negative
Fair E	465	2,568	H1N1		434	2,525	Negative
Fair F	286	1,339	Negative		325	2,099	Negative
Fair G	597	3,813	Negative		659	3,258	Negative
Fair H	523	3,115	H3N2		593	3,549	Negative
Total	3,313	19,561			3,455	20,207	

*The number of swine exhibited at fair refers to the total number of individual pigs enrolled in the study. The number of samples collected is the total number of nasal wipes collected during the entire fair. The HA-NA subtypes we isolated are included for the IAV-positive fairs. HA, hemagglutinin; IAV, influenza A virus; NA, neuraminidase.

**Figure 1:**
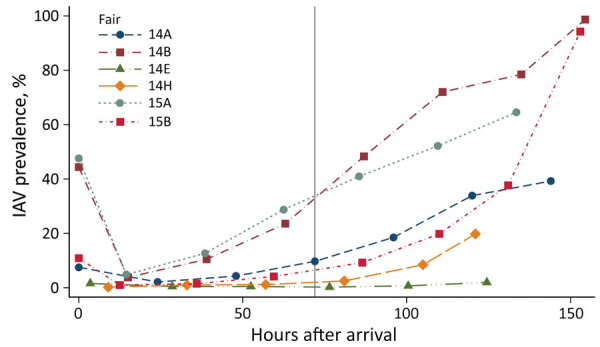
Percentage of pigs that tested positive for IAV by real-time reverse transcription PCR at 6 IAV-positive agricultural fairs, Ohio and Indiana, USA, 2014 and 2015. Each data point represents the prevalence at that sampling timepoint connected with colored lines to indicate trend over time for each individual fair. IAV prevalence rises steeply through the latter half of each fair, indicating the strong role of lengthy show duration in increased viral amplification in each swine population. The reference line shows the recommended 72-hour cutoff for swine show duration. IAV, influenza A virus.

We recovered IAV isolates from swine at 4 fairs in 2014 and 2 fairs in 2015, for a total of 6 IAV–positive fair events, at which we collected 15,162 samples; of those, 2,514 (16.6%) tested positive for IAV by rRT-PCR across all sampling times. Examining the rRT-PCR prevalence at each fair over time, we observed a clear increasing trend, providing strong evidence that these fair events had active IAV outbreaks ongoing in the exhibition swine population (Figure 1). IAV prevalence in all 6 fairs was relatively low through the first several 24-hour timepoints but increased dramatically by the end of the fair events as the outbreak spread throughout the swine barn. The exception to this trend was fair 2014 E. Although fair 2014 E did have an increasing trend in prevalence, it ended with only 6 rRT-PCR–positive samples of the 309 collected on the last day.

**Figure 2:**
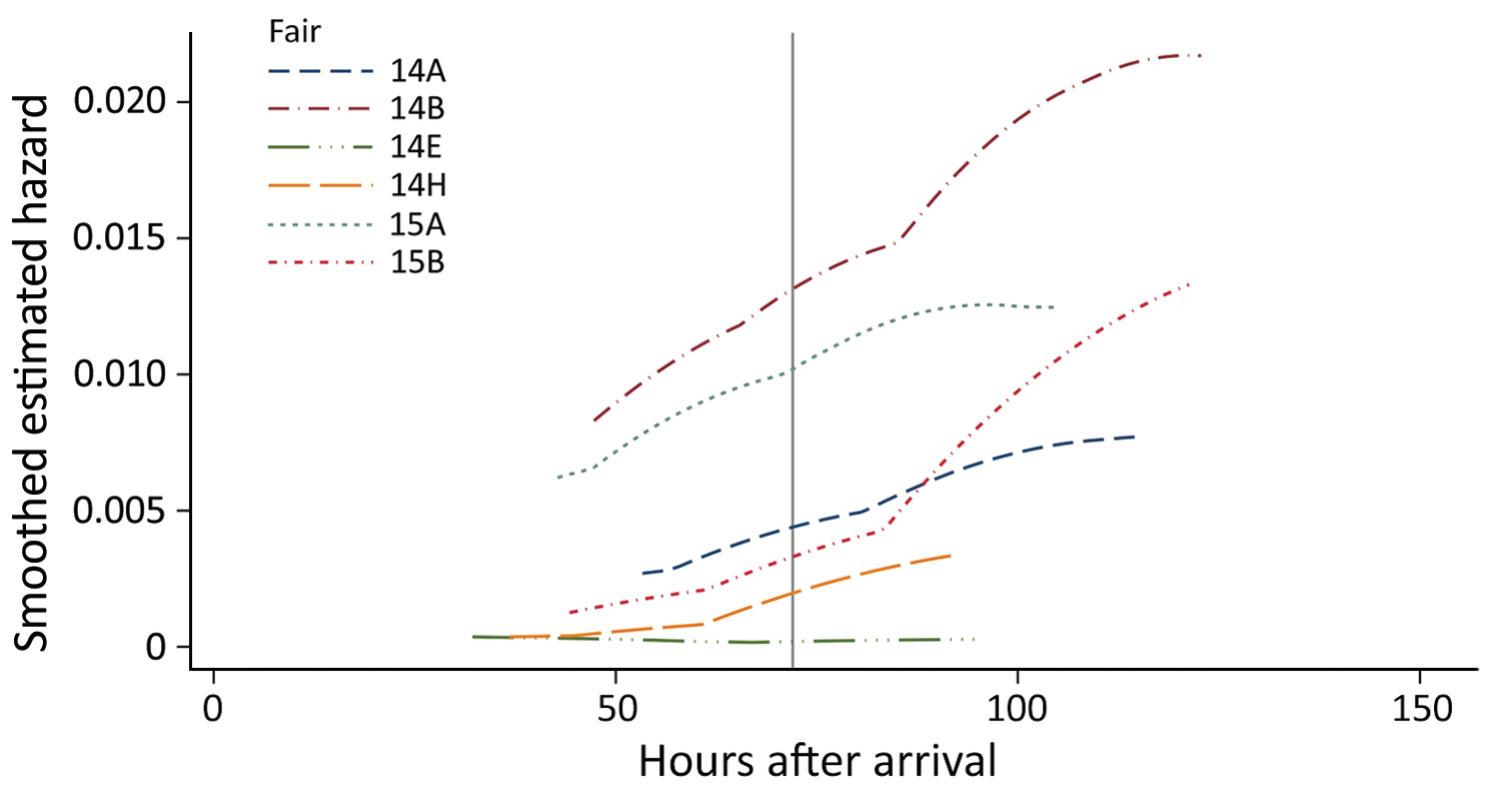
Estimated smoothed hazard of IAV infection over the number of hours since the origin of the fair for individual pigs at risk at 6 IAV-positive agricultural fairs, Ohio and Indiana, USA, 2014 and 2015. All 6 IAV-positive fairs from our longitudinal study are shown individually. Overall, hazard estimates increase throughout the duration of the fair. The exceptionally low hazards for fair 14E correspond to the low incidence of IAV documented in Figure 1 and Appendix Table 1 (https://wwwnc.cdc.gov/EID/article/28/10/22-0649-App1.pdf). IAV, influenza A virus.

By the end of fair 2014 B, we observed the highest IAV prevalences, 98.7% by rRT-PCR and 77.5% by virus isolation (Figure 1; Appendix Table 1). Also at fair 2014 B, using rRT-PCR, we detected IAV in only 16 (3.8%) of samples 15 hours after arrival at the second sampling, which highlights how critical the duration of swine shows can be as a factor in an IAV outbreak at any individual county fair. As expected in a growing outbreak, the estimated hazard increased with time (Figure 2). Visible in the increasing prevalence (Figure 1) and hazard estimates (Figure 2), very little leveling off occurred. The risk for IAV infection in swine continued to rise throughout the duration of the fairs. Given enough time, we found that most of the swine at a fair become infected with IAV, resulting in high viral load within the barn that could lead to zoonotic transmission to humans. These data provide strong support for the recommendation to limit the amount of time swine spend at a fair to curtail the interspecies transmission of IAV at county fairs.

### Acceptance of the 72-hour Limitation

We collected surveillance samples from 195 county fairs during the summers of 2018 and 2019. Based on the date of entry for swine, 144 of those fairs had not released swine before 72 hours. We sampled 38 fairs whose organizers stated that they released a portion of their swine before 72 hours onsite. In addition, 13 of the fairs at which we sampled had fully implemented the 72-hour recommendation and released all of their swine in <72 hours. Although the recommendation to shorten swine exhibitions had been around for >5 years, the adoption of this recommendation was still quite limited at the county fairs at which we collected samples. We compiled and categorized additional characteristics for the 195 county fairs sampled, including state, size, month, and IAV vaccine requirement (Appendix Table 2).

Among 2018 and 2019 county fairs that tested positive for IAV, those that did not apply the 72-hour recommendation had the highest average estimated prevalence (Table 2). The overall Kruskal–Wallis test (p = 0.0425) indicated that estimated prevalence is not the same across fairs that applied the 72-hour recommendation at the 3 different levels. Pairwise tests revealed that fairs that released all pigs before 72 hours had significantly lower prevalence estimates compared with fairs that did not release any pigs (p = 0.0176) (Table 2). Although fairs that released some pigs before 72 hours had lower prevalence estimates on average, we did not detect a significant difference between those fairs and fairs that did not release any pigs early (p = 0.3346) (Table 2). In addition, the only other fair characteristic found to be associated with IAV prevalence among IAV-positive fairs was that Indiana fairs had lower IAV prevalence compared with those in Ohio (Appendix Table 3).

**Table 2:** Number of county fairs sampled and number of fairs with swine testing positive for IAV, by categorical level at which the 72-hour recommendation was implemented, Ohio, Indiana, and Michigan, USA, 2018 and 2019*

Categorical level of implementation of 72-hour recommendation	Total no. fairs	No. IAV-positive fairs	Mean estimated IAV prevalence, %†	p value‡
No implementation	144	26	40.9	Referent
Some swine released	38	10	33.2	0.3346
All swine released	13	3	6.1	0.0176

*Number of county fairs sampled and number of fairs that tested IAV-positive in 2018 and 2019 are shown by the categorical level at which the 72-hour recommendation was implemented. IAV, influenza A virus. †Mean estimated IAV prevalence among the IAV-positive county fairs was estimated by using virus isolation data. ‡By Dunn’s test. Pairwise comparisons were completed by using “no implementation” as the reference category.

## Discussion

Through 2 active IAV field surveillance efforts, we found strong evidence that shortening the duration of swine exhibitions greatly reduces IAV prevalence in swine. In our longitudinal study sampling every pig every day, the IAV prevalence and hazard estimate at each IAV-positive fair rose consistently with increasing time at the fair, as we expected in the case of a growing infectious disease outbreak. Accordingly, if fairs had shortened their swine exhibitions, the maximum prevalence by the end of the fair would have been greatly reduced. For example, fair 2014 H and fair 2015 B displayed relatively parallel growth curves (Figure 1), and both had a PCR prevalence of 19.8% at the sixth sampling interval (121 hours after arrival at fair 2014 H and 110 hours after arrival at fair 2015 B). A critical difference between the 2 fairs, however, is that 2014 H ended after that sampling, resulting in the second-lowest end-of-fair prevalence in our study. In stark contrast, 2015 B had pigs on site through 153 hours and ended with 94.2% IAV PCR prevalence, the study’s second-highest peak prevalence. Despite a rise in IAV-positive samples through the first 6 days, in ending ≈2 days earlier, IAV prevalence at 2014 H never exceeded 20%, greatly reducing the viral load in the barn at the end of the fair and reducing the risk for zoonotic transmission.

Among the 6 fairs that tested IAV-positive, we found considerable variation in the rate at which the outbreaks grew. Only fair 2014 E maintained very low IAV prevalence, never exceeding 2% prevalence. The reason this fair’s prevalence remained so low compared with the other IAV-positive shows is unclear, but identification of management practices within fairs that can effectively flatten IAV spread in the swine barn should be an area of ongoing research. Reducing the transmission rate of IAV within fairs is a mitigation strategy for keeping IAV prevalence low, in addition to reducing the duration of fairs. A previous survey of swine exhibitors at jackpot shows found that most of those exhibitors supported many of the recommendations to minimize IAV risk (*24*). However, an advantage of mitigation measures like the 72-hour cutoff is that they can be applied at the fair level and do not require individual exhibitor compliance. For example, the effectiveness of the recommendation to not show any pigs with clinical signs of IAV infection is highly dependent on individual exhibitor education, awareness, and acceptance. As a result, we expect that, to varying degrees, pigs are likely to arrive infected with IAV (*14*). Therefore, recommendations that limit spread of IAV once it has been introduced to a county fair and can be put in place by fair organizers is a critical step toward reducing IAV prevalence in swine and the risk for zoonotic transmission of IAV.

Another control measure commonly recommended is vaccinating swine against IAV. Fair B tested positive in both 2014 and 2015 and had extraordinarily high peak IAV prevalence (98.7% in 2015 and 94.2% in 2015). Despite similarly high viral loads by the end, in 2015 the growth curve was flatter, crossing the 72-hour threshold at <10% IAV prevalence compared with >30% in 2014. In 2015, fair B added a required influenza vaccine for swine. Although we can only speculate as to whether the slower spread was attributable to vaccination, this finding would be consistent with previous work demonstrating that vaccination against IAV resulted in a shorter period of virus shedding and lower peak nasal titers in swine challenged with a heterologous IAV (*25*). Combining vaccination against IAV with reduced show duration could prove to be highly effective in reducing the risk for zoonotic transmission of IAV.

In all 4 fair events with a weigh-in upon arrival, the first sampling timepoint had a much higher PCR prevalence than did the second timepoint. Infected swine deposit IAV onto shared surfaces during these weigh-in events, which can lead to mass exposure of noninfected swine as they arrive (*26*). Because the nasal wipe samples primarily collected nasal secretions from the exterior of the nostrils, we detected numerous contaminations through PCR upon first sampling. It is improbable that all of those swine were actively shedding IAV while arriving at the fair, especially considering the drastic drop in prevalence for the following days, when we sampled the same pigs at their individual pens in the barn. However, this pattern illustrates that >50% of the pigs at some fairs are exposed to IAV almost immediately after arrival. In these cases, ending the fair before 72 hours is not necessarily preventing IAV infection in exposed pigs. Rather, by not affording ample time for infection and amplification, the shortening eliminates the concentration of swine at the fair and thereby does not expose fairgoers to the high IAV viral load that could be present if the pigs remained in the barn. In addition to shortening the time pigs are at the fair, we recommend that fair organizers alter the structure of their weigh-in events to reduce infectious disease transmission. In addition to the washing and disinfecting procedures previously recommended (*26*), using owner-declared pig weights or open scales available for use in the barn over longer periods of time could also limit the effect of weigh-in on IAV transmission.

In our investigation in 2018 and 2019, we found extremely limited implementation of the 72-hour rule. We detected IAV at only 3 county fairs that had fully implemented the 72-hour rule in 2018 and 2019, which reduced our statistical power to detect differences. However, the downward trend in prevalence at fairs that release swine before 72 hours (Table 2) provides strong evidence that the recommendation results in fewer swine actively shedding IAV by the end of a fair and therefore reduces the risk for zoonotic IAV transmission. Although we saw a dramatic decrease in IAV prevalence for the 3 IAV-positive fairs that released all swine before 72 hours, the effect was less pronounced and not statistically significant for the 10 fairs that only released some pigs. We did not have enough IAV-positive fairs in this sample to build a model adjusting for other risk factors associated with fair prevalence. However, we did not identify any additional risk factors associated with IAV prevalence (Appendix Table 3). The only exception was fairs in Indiana, which had significantly lower IAV prevalence compared with Ohio despite a high proportion of fairs testing IAV-positive in Indiana (*18*). Because Indiana encouraged its county fairs to implement the 72-hour recommendation compared with other states, the 72-hour swine release is more proximate in the causal pathway explaining IAV prevalence in this system.

Although we do expect population size and density to play a role in infectious disease outbreak growth (*27*), we also expect contact network structure to strongly influence transmission dynamics (*28*). On the basis of the findings at weigh-in, many arriving swine are exposed to IAV early during a fair. If swine are often infected before 72 hours but do not begin actively shedding virus until closer to the end of the fair, then reducing the population density will have limited influence on outbreak spread. Because aerosolized IAV is shed into the air at county fairs (*29*), the reduced number of swine shedding IAV in the barn still reduces the overall viral load and therefore zoonotic transmission risk associated with that fair. Animals that return home early may already be exposed to IAV and introduce infection to the home farm. These animals are still infected but are no longer on public display, which reduces public health risk at the fair but can still result in pathogen dissemination and complicates home–farm transmission and downstream network implications when swine attend additional shows (*13*,*18*). Although our study is limited to IAV surveillance data, other agriculturally relevant pathogens have been detected in exhibition swine and are probably also affected by these transmission mechanisms (*30*,*31*).

Culturally, agricultural exhibitions are imperative to attracting the interest of and training the next generation of agriculturalists, upon whom we rely for safe and secure food sources. However, contracting IAV from swine at agricultural exhibitions not only poses a local and global public health risk but could also deter youth exhibitors and undermine the public image of and confidence in agriculture and food production. Because IAV is consistently introduced to county fairs from upstream sources (*13*–*15*,*17*,*18*), it is imperative that we provide measures to reduce IAV risk within an individual fair. Shortening the duration of swine exhibitions at county fairs reduces IAV prevalence in exhibition swine and the subsequent risk for harmful zoonotic emergence.

Appendix Additional information on shortening duration of swine exhibitions to reduce risk for zoonotic transmission of influenza A virus.
