# Supplementary material for: Shortening Duration of Swine Exhibitions to Reduce Risk for Zoonotic Transmission of Influenza A Virus
Source: Emerg Infect Dis. 2022 Oct;28(10):2035–42. doi: 10.3201/eid2810.220649 (PMC9514346; doi:10.3201/eid2810.220649)
Supplement: Appendix — Additional information on shortening duration of swine exhibitions to reduce risk for zoonotic transmission of influenza A virus. [file 22-0649-Techapp-s1.pdf]

Article DOI: https://doi.org/10.3201/eid2810.220649

Shortening Duration of Swine Exhibitions to Reduce Risk for Zoonotic Transmission of Influenza A Virus

Appendix

Appendix Table 1. Influenza A virus (IAV) prevalence by PCR in exhibition swine is shown for each of the six IAV positive fairs in our longitudinal study*

	IAV prevalence (no. of samples)							
Fair	Sampling 1	Sampling 2	Sampling 3	Sampling 4	Sampling 5	Sampling 6	Sampling 7	Sampling 8
2014 A	7.5% (359)	2.1% (380)	4.3% (372)	9.7% (289)	18.5% (189)	33.9% (180)	39.2% (158)	
2015 A	47.6% (395)	4.8% (393)	12.7% (379)	28.7% (334)	40.9% (232)	52.2% (184)	64.6% (175)	
2014 B	44.4% (419)	3.8% (421)	10.5% (420)	23.6% (420)	48.3% (416)	72.0% (336)	78.5% (158)	98.7% (151)
2015 B	10.9% (414)	1.0% (411)	1.5% (410)	4.1% (411)	9.2% (444)	19.8% (328)	37.7% (162)	94.2% (139)
2014 E	1.6% (445)	0.4% (466)	0.4% (459)	0.2% (457)	0.7% (432)	1.9% (309)		
2014 H	0.2% (523)	1.0% (521)	1.2% (519)	2.5% (521)	8.5% (520)	19.8% (511)		

*The total number of samples collected for each time point is shown in parentheses. The first sampling time point occurred during either arrival or the first night after arrival with each subsequent time point occurring overnight at 24-h intervals until all swine left the fair.

Appendix Table 2. Additional fair characteristics for the 195 county fairs sampled during 2018 – 2019 are shown by the categorical level at which the 72-h recommendation was implemented*

Characteristic		Number of fairs	No implementation	Some swine released	All swine released
State					
	Indiana	71	40	25	6
	Michigan	29	28	1	0
	Ohio	95	76	12	7
Fair size					
	>200 pigs	82	50	27	5
	≤200 pigs	75	61	7	7
Fair month of sampling					
	June	18	14	3	1
	July	90	54	29	7
	August	57	49	4	4
	September	26	23	2	1
	October	4	4	0	0
Influenza vaccine required					
	Yes	25	18	7	0
	No	126	88	28	10

*There are some missing data for vaccine requirement and number of pigs at each fair where this information could not be obtained or was not known at the time of sampling.

Appendix Table 3. Mean estimated influenza A virus (IAV) prevalence among IAV positive county fairs sampled during 2018 – 2019 is shown by categorical fair characteristics*

Fair characteristic		Number of IAV positive fairs	Mean estimated IAV prevalence (positive fairs)	Overall p-value (Kruskal-Wallis)	Pairwise p-value (Dunn's Test)
State					
	Indiana	25	27.2%	0.0231	-
	Michigan	5	47.4%		0.0820
	Ohio	9	55.0%		0.0173
Fair size					
	>200 pigs	21	34.3%	0.5232	
	≤200 pigs	7	47.9%		
Fair month of sampling					
	June	5	38.3%	0.2483	
	July	26	29.6%		
	August	7	58.9%		
	September	1	40.0%		
	October	0	-		
Influenza vaccine required					
	Yes	6	33.9%	0.9425	
	No	23	36.0%		

*Partially due to the overall low number of fairs testing positive for IAV during this time frame, the only significant different identified was Indiana fairs having lower IAV prevalence compared to Ohio (and marginal evidence for a difference compared to Michigan). There are some missing data for vaccine requirement and number of pigs at each fair where this information could not be obtained or was not known at the time of sampling.
